# The role of the hemoglobin-albumin-lymphocyte-platelet (HALP) score in deep neck infections and its relationship with clinical parameters

**DOI:** 10.55730/1300-0144.6024

**Published:** 2025-03-15

**Authors:** Ayşe Seçil KAYALI DİNÇ, Nagihan GÜLHAN YAŞAR, Agah YENİÇERİ, Çetin ŞAHUTOĞLU, İbrahim SEVİM, Melih ÇAYÖNÜ

**Affiliations:** Department of Otorhinolaryngology, Head, and Neck Surgery, Ankara Bilkent City Hospital, Ankara, Turkiye

**Keywords:** Deep neck infections, HALP score, abscess, severity, treatment

## Abstract

**Background/aim:**

Deep neck infections (DNI) are suppurative infections of the cervical spaces, which may lead to life-threatening complications. The HALP score is a laboratory parameter reflecting a patient’s nutritional and inflammatory status. The aim of this study was to compare the HALP scores of patients with DNI with those of a control group. A secondary objective was to investigate the potential relationship between the HALP score and clinical parameters, and to assess its role in determining disease severity.

**Materials and methods:**

The study retrospectively included patients who were hospitalized and treated for DNI. Data were extracted from the hospital’s electronic records. The control group comprised patients admitted for surgery with a diagnosis of septal deviation. Patients with DNI were designated as Group 1, and the control subjects as Group 2. HALP scores were calculated using a defined formula. The HALP scores of patients with DNI were compared with those of the control group, and the relationship between the HALP score and various clinical parameters was subsequently evaluated.

**Results:**

A total of 433 patients were included in the study. The groups were similar in terms of number of patients, age, and sex. Hemoglobin, albumin, lymphocyte count, and HALP scores were significantly lower in Group 1, whereas leukocyte, neutrophil, monocyte, and platelet counts were significantly higher. A negative correlation was observed between the HALP score and length of hospital stay (r = −0.293, p < 0.01), abscess dimensions (height: r = −0.271; length: r = −0.267, p < 0.01), and CRP levels (r = −0.222, p < 0.01).

**Conclusion:**

Patients with DNI exhibited lower HALP scores than the normal population. HALP scores below 46.14 were significantly associated with indicators of severe disease, including an elevated inflammatory response, larger abscess size, and prolonged hospital stay.

## 1. Introduction

Deep neck infections (DNI) are suppurative infections that develop within the potential spaces filled with loose connective tissue between the three layers of the deep cervical fascia.

Although DNI do not typically begin as cellulitis, if left untreated, they may progress to form abscesses, extend along the neck, and eventually spread to the mediastinum [1]. DNI may lead to serious complications such as airway obstruction, mediastinitis, jugular vein thrombophlebitis, cranial nerve dysfunction, cervical osteomyelitis, meningitis, and even death [2].

In cases of DNI, early assessment of disease severity and prompt selection of appropriate treatment not only reduce the risk of severe complications but also help prevent prolonged hospitalization [3]. Therefore, there is a need for easily calculable scores in clinical practice to determine the severity of DNI. One such score, which may be useful for this purpose, is the Hemoglobin-Albumin-Lymphocyte-Platelet (HALP) score. This score is important because it provides information about the patient’s inflammatory status, nutritional status, and thromboembolic risk. In DNI patients, these parameters are closely related to the course of the disease. DNI is a life-threatening emergency condition. The condition may begin with a minor abscess and progress to more extensive infections. Identifying an index that can determine disease severity at an early stage will facilitate the management of DNI. In this context our study may shed light on this issue in the literature.

The HALP score, a laboratory parameter reflecting nutritional and inflammatory status, was first described in 2015. It is calculated as [hemoglobin (g/L) × albumin (g/L) × lymphocyte count (/L)] / platelet count (/L) [4].

The primary aim of the present study was to compare the HALP scores of patients admitted with DNI with those of a control group. The secondary aim was to investigate the potential association between the HALP score and clinical parameters, thereby determining its role in assessing the severity of the disease.

## 2. Materials and methods

The study included all male and female patients aged 18–65 years who presented to the emergency department with a DNI and were subsequently hospitalized for treatment between April 2019 and October 2024. Patient parameters at the time of admission were recorded.

Patients out of the specified age range of 18–65 years, those who refused hospitalization, those with incomplete or inaccessible records, and those with incomplete blood results were excluded.

Data collected for each patient included demographic information (first name, last name, age, sex), abscess characteristics (location and dimensions), abscess drainage status, complete blood count (hemoglobin, white blood cell count, neutrophils, lymphocytes, monocytes, platelets), total protein, albumin, fibrinogen, C-reactive protein (CRP), and length of hospital stay. All blood counts used in this study were obtained at the time of the patient’s initial hospital admission.

The control group was formed by including patients admitted for surgical treatment due to a diagnosis of septal deviation. Patients with DNI were designated as Group 1, while the control group was designated as Group 2.

Using the collected parameters, the HALP score for each patient was calculated as follows. The patient’s hemoglobin, albumin, and lymphocyte count were multiplied, and the result was divided by the patient’s platelet count.

Formula:


[hemoglobin (g/L)×albumin (g/L)×lymphocyte count (/L)]/platelet count (/L)

The HALP scores of patients with DNI were compared with those of the control group, and the relationship between the HALP score and various clinical parameters was subsequently evaluated.

## 3. Statistical analysis

All statistical analyses were performed using SPSS version 1.0.0.1508 for macOS (SPSS Inc., Chicago, IL, USA). The one-sample Kolmogorov–Smirnov test was used to assess the normality of data distribution. Since all variables did not follow a normal distribution, nonparametric tests were applied in the analysis.

Continuous variables were presented as mean ± standard deviation (SD), and group comparisons were conducted using the Mann–Whitney U test. Categorical variables were expressed as frequencies and percentages and analyzed using the chi-square test.

Correlations between clinical and laboratory parameters were evaluated using Spearman’s correlation coefficient. The diagnostic performance of HALP in predicting DNI severity was assessed using receiver operating characteristic (ROC) curve analysis. A p-value <0.05 was considered statistically significant.

## 4. Results

The mean age of Group 1was 36.61 ± 11.35 years (range: 19–65), while Group 2 had a mean age of 33.29 ± 10.96 years (range: 18–64). Sex distribution was similar between the groups. There were 73 females (34.3%) and 140 males (65.7%) in Group 1, and 75 females (34.1%) and 145 males (65.9%) in Group 2.

In Group 1, hemoglobin, albumin, lymphocyte counts, and the HALP score were significantly lower compared with Group 2. Conversely, leukocyte, neutrophil, monocyte, and platelet counts were significantly higher in Group 1. Total protein levels showed no significant difference between the groups, while CRP data were available only for Group 1, with a mean value of 120.43 ± 90.33 (Table 1).

The mean hospitalization duration of Group 1 was 7.82 ± 5.96 days (range: 1–55 days). The mean abscess size was 25.02 ± 13.69 mm in height and 20.86 ± 11.59 mm in length. Regarding drainage status, 69.5% (n = 148) of DNI patients underwent drainage, while 30.5% (n = 64) did not require any invasive procedure. There was no statistically significant difference in HALP scores between the group with abscess drainage and the group without it.

The most common abscess location was peritonsillar, observed in 144 patients (67.6%), followed by the submandibular region in 32 patients (15%). Parapharyngeal abscess was identified in 23 patients (10.8%), while retropharyngeal abscess was detected in six (2.8%). Submental/Ludwig’s angina was noted in five patients (2.3%), while multiple-site infections were present in two patients (0.9%). The least common abscess type was parotid abscess, observed in only one patient (0.5%) (Table 2).

The correlation analysis identified significant associations between clinical parameters in DNI patients. A strong positive correlation was observed between age and length of hospital stay (r = 0.238, p < 0.01), as well as between hospital stay and abscess size (r = 0.348 for height, r = 0.302 for length, p < 0.01), indicating that larger abscesses and older age were associated with prolonged hospitalization. Additionally, higher CRP levels correlated with both the length of hospital stay (r = 0.190, p < 0.01) and abscess size (r = 0.134 for height, p = 0.051; r = 0.147 for length, p < 0.05). In contrast, the HALP score demonstrated significant negative correlations with hospital stay (r = −0.293, p < 0.01), abscess size (r = −0.271 for height, r = −0.267 for length, p < 0.01), and CRP levels (r = −0.222, p < 0.01). These findings indicate that lower HALP scores were associated with greater inflammation, larger abscesses, and prolonged hospitalization (Table 3).

The ROC analysis for the HALP score in predicting DNI severity showed an area under the curve (AUC) of 0.754 (95% CI: 0.708–0.799, p < 0.001), indicating moderate to good diagnostic accuracy. The optimal cut-off was set at HALP ≤46.14, achieving 70% sensitivity and 64% specificity (Figure).

## 5. Discussion

Our study yielded two key findings. The first is that the HALP score was lower in the patient group with DNI. The second is the observed relationship between a HALP score below 46.14 and disease severity.

In recent years, a novel prognostic biomarker known as HALP has emerged in the literature for predicting a range of clinical outcomes [4]. This immunonutritional marker is derived from the integration of parameters reflecting immune status (lymphocyte and platelet counts), nutritional status (albumin), and anemia status (hemoglobin) [5]. Although it was initially developed as a scoring system for cancer patients [6], subsequent studies have demonstrated the utility of the HALP score in noncancer conditions as well [7,8,9]. To the best of our knowledge, despite the proven efficacy of the HALP score in various diseases, no study has yet evaluated its effectiveness in patients with DNIs, who are prone to significant complications.

Among the parameters used in the HALP score, the platelet count plays a critical role. Platelets secrete vascular endothelial growth factor (VEGF), which triggers the release of inflammatory mediators [10]. VEGF, one of the proangiogenic molecules, not only functions as a mitogen and angiogenic factor specific to endothelial cells but also significantly increases vascular permeability. Upon binding to its receptor, VEGF activates intracellular signaling pathways that lead to inhibition of apoptosis, capillary dilation, and increased permeability [11]. This cascade is a key component of the inflammatory process. In patients with DNI, the clinical spectrum may range from mild inflammation to sepsis. In the present study, the DNI patient group exhibited elevated platelet counts. These increased platelet levels may contribute to the heightened inflammatory state observed in DNI, and, in turn, lead to a reduction in the HALP score. This finding demonstrates that a low HALP score is closely associated with an intensified inflammatory process.

Another component of the HALP score is the albumin level. Albumin levels are closely related to nutritional status, and both inflammation and poor nutritional status have been associated with low albumin levels [12]. Serum albumin is the most abundant protein in circulation. It acts as a negative acute-phase protein, decreasing in response to inflammation. It inhibits adhesion molecules on endothelial cells and exhibits antiinflammatory effects at physiological concentrations [13]. Furthermore, serum albumin possesses multiple binding sites and exhibits antioxidant activity due to its free radical scavenging properties [14]. In the present study, albumin levels were low in patients with DNI, and these reduced levels were associated with severe inflammation.

HALP is an inflammatory marker that reflects the clinical and nutritional status of the patient. This easily calculable parameter provides information about the severity of DNI and can be useful in the clinical treatment process.

Since all blood counts were obtained at the time of initial hospital admission, the lower hemoglobin levels in DNI patients were not influenced by intravenous fluid administration or blood loss from surgical intervention. Many DNI patients, particularly those with poor oral intake, dysphagia, or chronic inflammation, may have iron, folate, or vitamin B12 deficiencies, impairing red blood cell (RBC) production. Albumin levels were also lower in DNI patients in this study, suggesting compromised nutritional status, which likely contributes to reduced hemoglobin synthesis. Lower hemoglobin levels reduce oxygen-carrying capacity, leading to poor tissue oxygenation and delayed wound healing, and may indicate prolonged hospital stays and slower recovery. Lymphopenia is a hallmark of severe infections, indicating immune suppression or excessive inflammatory response. Moreover, lymphopenia is associated with poorer outcomes in severe infections, including sepsis, pneumonia, and deep tissue infections.

Our findings suggest that DNI patients exhibit significantly lower HALP scores compared with controls, correlating with higher CRP levels, larger abscess size, and prolonged hospitalization. This aligns with prior research showing that malnutrition and systemic inflammation contribute to worse infectious disease outcomes. However, unlike studies in oncology where HALP is a strong predictor of survival, our results indicate that HALP provides only moderate predictive power in DNI (AUC = 0.754, sensitivity 70%, specificity 64%). The ability to rapidly assess disease severity in DNI is critical, as these infections can progress to life-threatening complications like airway obstruction and mediastinitis. Current DNI risk stratification relies on imaging (CT scan, ultrasound) and broad inflammatory markers (CRP, white blood cell (WBC) count). HALP offers a simple, cost-effective alternative that could help in early risk assessment and treatment decision-making. However, due to its moderate predictive accuracy, HALP should be used alongside other established severity indices rather than as a standalone marker.

The main limitation of our study is its retrospective design.

## 6. Conclusion

Patients presenting to the hospital with DNI exhibited significantly lower HALP scores compared with the normal population. HALP scores below 46.14 were significantly associated with indicators of severe disease, including an elevated inflammatory response, larger abscess size, and prolonged hospital stay. The HALP score, being both simple to calculate and practical, can be effectively used in clinical settings to assess the severity of DNI.

## Figures and Tables

**Figure:**
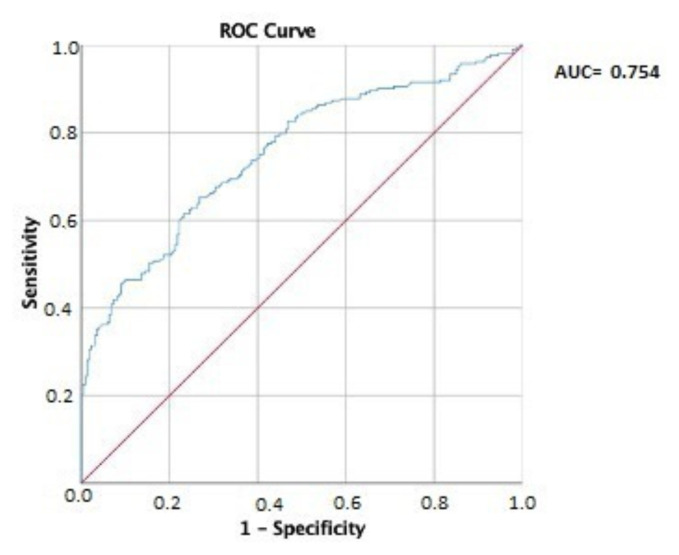
The ROC analysis for HALP score in predicting DNI severity. AUC: Area under the curve.

**Table 1:** Comparison of hematological parameters between groups.

	Group 1 (n=213 )	Group 2(n=220)	p
**Hemoglobin**	14.15±1.77	14.82±1.45	**<0.001**
**Total protein**	72.07±5.88	72.39±3.86	0.502
**Albumin**	44.06±3.88	46.72±2.53	**<0.001**
**Leukocyte**	14.15±5.66	7.26±1.79	**<0.001**
**Neutrophil**	11.09±5.52	4.31±1.46	**<0.001**
**Lymphocyte**	1.95±1.01	2.17±0.59	0.005
**Monocyte**	0.82±0.47	0.41±0.12	**<0.001**
**Platelet**	295.67±98.1	249.03±53.45	**<0.001**
**HALP score**	43.82±22.87	62.95±22.26	**<0.001**

p < 0.05 is statistically significant.

**Table 2:** Distribution of abscess locations in patients with DNI.

Abscess location	n	%
**Submandibular**	32	15.0
**Peritonsillar**	144	67.6
**Parapharyngeal**	23	10.8
**Submental/Ludwig’s**	5	2.3
**Multiple sites**	2	0.9
**Retropharyngeal**	6	2.8
**Parotid**	1	0.5
**Total**	213	100

**Table 3:** Correlation analysis of clinical parameters.

	Age (r / p)	Length of hospital stay (r / p)	Abscess height (mm) (r / p)	Abscess length (mm) (r / p)	CRP (r / p)	HALP (r / p)
**Age**		0.238^**^ / p < 0.001	0.089 / 0.194	0.101 / 0.143	0.074 / 0.281	−0.081 / 0.093
**Length of hospital stay**	0.238^**^ / p < 0.001		0.348^**^ / p < 0.001	0.302^**^ / p < 0.001	0.190^**^ / 0.005	−0.293^**^ / p < 0.001
**Abscess height (mm)**	0.089 / 0.194	0.348^**^ / p < 0.001		0.807^**^ / p < 0.001	0.134 / 0.051	−0.271^**^ / p < 0.001
**Abscess length (mm)**	0.101 / 0.143	0.302^**^ / p < 0.001	0.807^**^ / p < 0.001		0.147^*^ / 0.032	−0.267^**^ / p < 0.001
**CRP**	0.074 / 0.281	0.190^**^ / 0.005	0.134 / 0.051	0.147^*^ / 0.032		−0.222^**^ / 0.001
**HALP**	−0.081 / 0.093	−0.293^**^ / p < 0.001	−0.271^**^ / p < 0.001	−0.267^**^ / p < 0.001	−0.222^**^ / p < 0.001	
